# Supplementary material for: Antimicrobial exposure is associated with decreased survival in triple-negative breast cancer
Source: Nat Commun. 2023 Apr 12;14:2053. doi: 10.1038/s41467-023-37636-0 (PMC10097670; doi:10.1038/s41467-023-37636-0)
Supplement: Supplementary file 1 — Supplementary Information [file 41467_2023_37636_MOESM1_ESM.pdf]

Supplemental Table 1: List of medications considered as antimicrobial exposures and growth factors and their administration routes.

Medication Category (% of patients)	Administration route
**Antibiotics (84%)**	
Amikacin	IV or injection
Amoxicillin	pill or tablet, oral suspension
Ampicillin	pill or tablet, oral suspension, IV or injection
Azithromycin	pill or tablet, oral suspension
Aztreonam	pill or tablet, oral suspension
Bacitracin	IV or injection, topical, ophthalmic
Cefaclor	pill or tablet
Cefadroxil	pill or tablet, oral suspension
Cefazolin	IV or injection
Cefdinir	pill or tablet
Cefepime	IV or injection
Cefixime	pill or tablet
Cefotaxime	IV or injection
Cefoxitin	IV or injection
Cefpodoxime	pill or tablet
Cefprozil	pill or tablet
Ceftaroline	IV or injection
Ceftazidime	IV or injection
Ceftriaxone	IV or injection
Cefuroxime	pill or tablet, oral suspension
Cephalexin	pill or tablet, oral suspension
Ciprofloxacin	IV or injection, pill or tablet, oral suspension
Clarithromycin	pill or tablet, oral suspension
Clindamycin	pill or tablet, IV or injection
Colistin	IV or injection
Dapsone	pill or tablet
Daptomycin	IV or injection
Dicloxacillin	pill or tablet
Doxycycline	pill or tablet, IV or injection
Ertapenem	IV or injection
Erythromycin	pill or tablet, IV or injection, oral suspension
Ethambutol	pill or tablet
Fidaxomicin	pill or tablet
Gentamicin	IV or injection
Imipenem	IV or injection
Isoniazid	pill or tablet
Levofloxacin	pill or tablet, IV or injection
Linezolid	pill or tablet
Meropenem	IV or injection
Methenamine	pill or tablet
Metronidazole	pill or tablet, IV or injection
Minocycline	pill or tablet
Moxifloxacin	pill or tablet, IV or injection
Nafcillin	IV or injection
Neomycin	pill or tablet
Nitrofurantoin	oral suspension, pill or tablet
Penicillin	oral suspension
Penicillin	pill or tablet

Medication Category (% of patients)	Administration route
Piperacillin	IV or injection
Polymyxin	IV or injection, inhalation, ophthalmic
Pyrazinamide	pill or tablet
Rifabutin	pill or tablet
Rifampin	pill or tablet
Rifaximin	pill or tablet
Streptomycin	IV or injection
Sulfamethoxazole	pill or tablet, oral suspension
Tetracycline	pill or tablet
Tobramycin	IV or injection
Trimethoprim	pill or tablet
Vancomycin	pill or tablet
**Antifungals (20%)**	
Amphotericin	oral suspension
Anidulafungin	IV or injection
Caspofungin	IV or injection
Clotrimazole	Topical, oral
Fluconazole	pill or tablet, oral suspension, IV or injection
Itraconazole	pill or tablet
Ketoconazole	pill or tablet
Nystatin	pill or tablet, oral suspension
Oxiconazole	topical
Posaconazole	oral suspension
Terbinafine	pill or tablet
Voriconazole	pill or tablet, IV or injection, oral suspension
**Growth factor support (41%)**	
Filgrastim	IV or injection, subcutaneous
Filgrastim	IV or injection, subcutaneous
Filgrastim	IV or injection
Filgrastim	IV or injection
Filgrastim	IV or injection
Filgrastim	IV or injection
Neupogen	IV or injection
Neupogen	IV or injection
Neupogen	IV or injection
Pegfilgrastim	IV or injection
Pegfilgrastim	IV or injection
Pegfilgrastim	Subcutaneous
Pegfilgrastim	IV or injection
Pegfilgrastim	IV or injection
Pegfilgrastim	IV or injection
Sargramostim	IV or injection, subcutaneous

Supplemental Figure 1: Flow diagram of patients meeting inclusion and exclusion criteria.

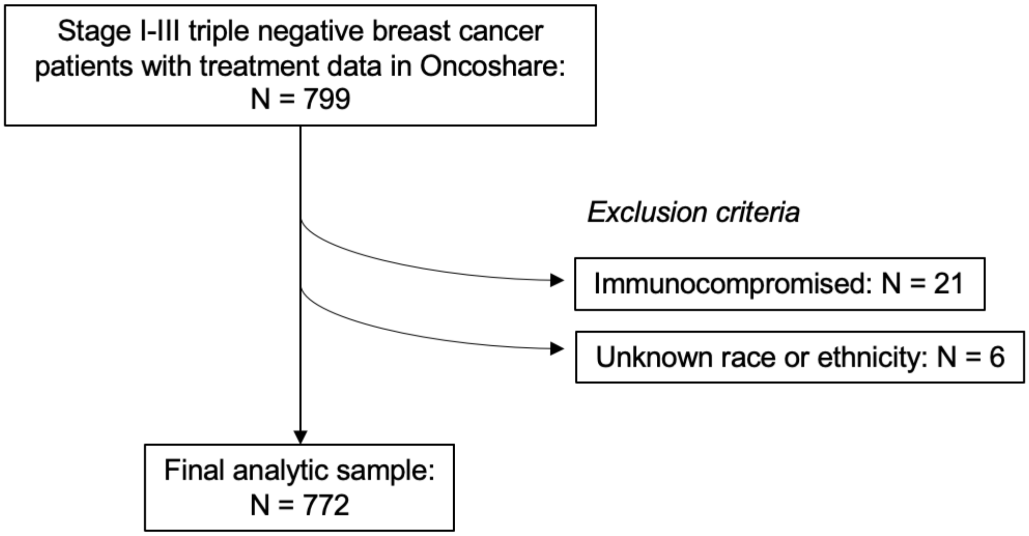

Supplemental Figure 2: Weighted distributions of total and unique antimicrobial prescriptions.

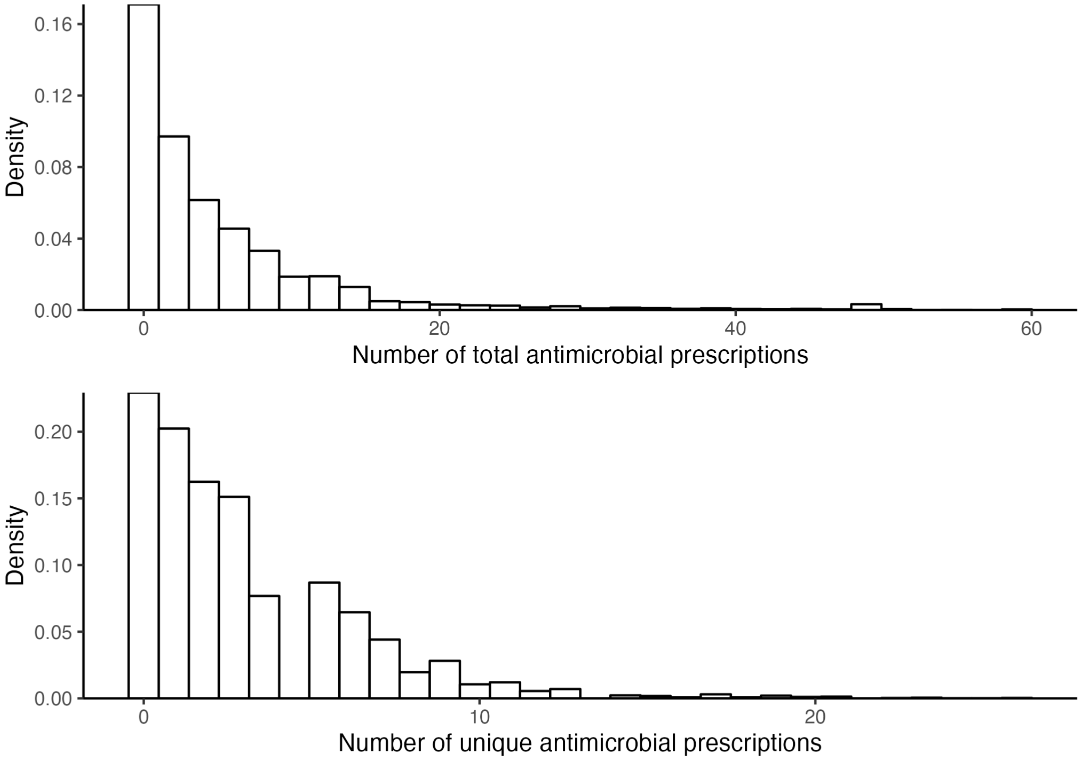

Supplemental Table 2: Inverse probability weighting results for any antimicrobial use.

Characteristic	Hazard Ratio (95% Confidence Interval)	p-value
Absolute lymphocyte count	0.88 (0.78 to 0.99)	0.040
Age at diagnosis	1.01 (1.00 to 1.01)	0.023
Race and ethnicity		
Non-Hispanic White	1.0	
Hispanic	0.93 (0.69 to 1.27)	0.66
Asian/Pacific Islander	0.84 (0.66 to 1.05)	0.12
Black/African American	0.85 (0.57 to 1.25)	0.41
Neighborhood socioeconomic status	1.00 (0.93 to 1.08)	>0.99
Stage		
I	1.0	
II	0.81 (0.68 to 0.98)	0.028
III	0.80 (0.61 to 1.04)	0.10
Tumor grade		
1	1.0	
2	1.48 (0.92 to 2.37)	0.11
3	1.36 (0.87 to 2.15)	0.18
Received chemotherapy		
No	1.0	
Yes	1.15 (0.91 to 1.46)	0.23
Received radiotherapy		
No	1.0	
Yes	0.97 (0.81 to 1.16)	0.76
Ever used growth factor support		
No	1.0	
Yes	1.90 (1.60 to 2.27)	<0.001
Surgery type		
Lumpectomy	1.0	
Bilateral Mastectomy	1.61 (1.25 to 2.08)	<0.001
Unilateral Mastectomy	1.52 (1.25 to 1.85)	<0.001

Hazard ratios were adjusted for all listed covariates and reflect the risk of death for any antimicrobial use, defined as ever versus never receipt of antimicrobials during the study period. For age at diagnosis, hazard ratio indicates the risk of death per one year of age increase. For neighborhood socioeconomic status, hazard ratio represents the risk of death per increase in each socioeconomic status quintile. Two-sided p-values were derived from Wald tests.

Supplemental Table 3: Inverse probability weighting results for total antimicrobial exposures.

Characteristic	Estimate (95% Confidence Interval)	p-value
Absolute lymphocyte count	0.02 (0.00 to 0.04)	0.015
Age at diagnosis	-0.01 (-0.02 to 0.00)	0.056
Race and ethnicity		
Non-Hispanic White	0.0	
Hispanic	-0.20 (-0.56 to 0.17)	0.29
Asian/Pacific Islander	-0.23 (-0.52 to 0.05)	0.11
Black/African American	-0.20 (-0.64 to 0.24)	0.37
Neighborhood socioeconomic status	0.05 (-0.05 to 0.15)	0.31
Stage		
I	0.0	
II	-0.10 (-0.39 to 0.19)	0.50
III	-0.62 (-1.0 to -0.22)	0.002
Grade		
1	0.0	
2	-0.32 (-0.95 to 0.31)	0.32
3	-0.52 (-1.2 to 0.12)	0.11
Unknown	-0.05 (-0.83 to 0.73)	0.90
Received chemotherapy		
No	0.0	
Yes	0.08 (-0.23 to 0.40)	0.61
Received radiotherapy		
No	0.0	
Yes	-0.02 (-0.29 to 0.25)	0.89
Ever used growth factor support		
No	0.0	
Yes	0.34 (0.02 to 0.66)	0.038
Surgery type		
Lumpectomy	0.0	
Bilateral Mastectomy	0.30 (-0.09 to 0.69)	0.14
Unilateral Mastectomy	0.30 (0.00 to 0.60)	0.049

Estimates were adjusted for all listed covariates and reflect the risk of death for each change in total number of per-month prescriptions. For age at diagnosis, estimate indicates the risk of death per one year of age increase. For neighborhood socioeconomic status, estimate represents the risk of death per increase in each socioeconomic status quintile. Two-sided p-values were derived from Wald tests.

Supplemental Table 4: Inverse probability weighting results for unique antimicrobial exposures.

Characteristic	Estimate (95% Confidence Interval)	p-value
Absolute lymphocyte count	0.01 (0.00 to 0.02)	0.072
Age at diagnosis	0.00 (-0.01 to 0.00)	0.50
Race and ethnicity		
Non-Hispanic White	0.0	
Hispanic	-0.01 (-0.26 to 0.23)	0.93
Asian/Pacific Islander	-0.14 (-0.34 to 0.05)	0.14
Black/African American	-0.03 (-0.35 to 0.30)	0.87
Neighborhood socioeconomic status	0.05 (0.00 to 0.11)	0.073
Stage		
I	0.0	
II	-0.10 (-0.25 to 0.04)	0.17
III	-0.32 (-0.53 to -0.10)	0.004
Grade		
1	0.0	
2	-0.09 (-0.44 to 0.26)	0.61
3	-0.11 (-0.45 to 0.23)	0.53
Unknown	0.11 (-0.32 to 0.53)	0.62
Received chemotherapy		
No	0.0	
Yes	0.04 (-0.16 to 0.25)	0.68
Received radiotherapy		
No	0.0	
Yes	0.01 (-0.15 to 0.17)	0.92
Ever used growth factor support		
No	0.0	
Yes	0.32 (0.17 to 0.46)	<0.001
Surgery type		
Lumpectomy	0.0	
Bilateral Mastectomy	0.34 (0.14 to 0.54)	<0.001
Unilateral Mastectomy	0.28 (0.11 to 0.45)	0.001

Estimates were adjusted for all listed covariates and reflect the risk of death for each change in unique number of per-month prescriptions. For age at diagnosis, estimate indicates the risk of death per one year of age increase. For neighborhood socioeconomic status, estimate represents the risk of death per increase in each socioeconomic status quintile. Two-sided p-values were derived from Wald tests.

Supplemental Table 5: Multivariable-adjusted hazard ratios for any, total, and unique antimicrobial exposures in the marginal structural Cox regression model.

Antimicrobial Exposure Definition	Overall Survival			Breast Cancer-Specific Survival		
	**N Events**	**Hazard Ratio (95% Confidence Interval)**	**p-value**	**N Events**	**Hazard Ratio (95% Confidence Interval)**	**p-value**
Any	171	1.46 (0.93 to 2.29)	0.10	124	1.39 (0.84 to 2.32)	0.20
Total	205	1.05 (1.02 to 1.08)	<0.001	142	1.05 (1.01 to 1.08)	0.005
Unique	200	1.17 (1.12 to 1.23)	<0.001	143	1.18 (1.13 to 1.24)	<0.001

Hazard ratios reflect the risk of death for any antimicrobial use, defined as ever versus never receipt of antimicrobials, and the risk of death for each change in cumulative number of per-month prescriptions for total and unique exposures during observation. Marginal structural Cox regression models were adjusted for the following variables: age at diagnosis, race and ethnicity, socioeconomic status quintile, cancer stage, tumor grade, receipt of chemotherapy, receipt of radiotherapy, ever use of growth factor support, and surgery type. Two-sided p-values were derived from Wald tests.

Supplemental Table 6: Marginal structural model adjusted for disease severity.

Antimicrobial Exposure Definition	Overall Survival			Breast Cancer-Specific Survival		
	N Events	Hazard Ratio (95% Confidence Interval)	p-value	N Events	Hazard Ratio (95% Confidence Interval)	p-value
Any	169	1.45 (0.93 to 2.28)	0.10	122	1.40 (0.84 to 2.34)	0.20
Acute illness		2.07 (1.24 to 3.46)	0.005		1.74 (0.90 to 3.36)	0.10
Total	218	1.05 (1.02 to 1.08)	<0.001	154	1.05 (1.02 to 1.09)	0.001
Acute illness		2.56 (1.42 to 4.62)	0.002		2.27 (0.99 to 5.17)	0.052
Unique	197	1.16 (1.11 to 1.21)	<0.001	142	1.17 (1.12 to 1.23)	<0.001
Acute illness		1.82 (1.09 to 3.02)	0.021		1.61 (0.82 to 3.16)	0.16

In addition to adjusting for acute illness, HRs were adjusted for all variables adjusted for in the marginal structural model, including: age at diagnosis, race and ethnicity, socioeconomic status quintile, cancer stage, tumor grade, receipt of chemotherapy, receipt of radiotherapy, ever use of growth factor support, and surgery type. Two-sided p-values were derived from Wald tests.

Supplemental Table 7: Unweighted versus marginal structural model adjusted hazard ratios to evaluate the impact of absolute lymphocyte versus neutrophil count on all-cause and breast cancer-specific survival related to any antimicrobial exposure.

**Characteristic**	**Overall Survival Model**		**Breast Cancer-Specific Survival Model**	
	**Unweighted**	**Marginal Structural**	**Unweighted**	**Marginal Structural**
Absolute lymphocyte count	1.54 (0.98 to 2.49)	1.46 (0.93 to 2.29)	1.47 (0.89 to 2.45)	1.39 (0.84 to 2.32)
Absolute neutrophil count	1.58 (1.01 to 2.49)	1.58 (1.01 to 2.48)	1.53 (0.92 to 2.54)	1.52 (0.92 to 2.53)

Results as shown as hazard ratio (95% confidence interval). Hazard ratios were adjusted for the following variables: age at diagnosis, race and ethnicity, socioeconomic status quintile, cancer stage, tumor grade, receipt of chemotherapy, receipt of radiotherapy, ever use of growth factor support, and surgery type.
